# Assessment of Impulse Noise Level and Acoustic Trauma in Military Personnel

**DOI:** 10.5812/kowsar.22517464.2674

**Published:** 2012-01-15

**Authors:** Maryam Rezaee, Mohammad Mojtahed, Mohammad Ghasemi, Babak Saedi

**Affiliations:** 1 Trauma Research Center, Baqiyatallah University of Medical Sciences, Tehran, IR Iran; 2 Department of Otolaryngology, Imam Khomeini Hospital, Tehran University of Medical Sciences, Tehran, IR Iran

**Keywords:** Hearing Loss, Auditory Fatigue, Military Personnel

## Abstract

**Background:**

Military personnel are usually exposed to high levels of impulse noise (IN) which can lead to hearing loss.

**Objectives:**

The purpose of this study was to assess the effects of relatively low level exposure of impulse noise (IN) during shooting practice on hearing using pure tone audiometry (PTA) and transiently evoked otoacoustic emission (TEOAE) in military personnel.

**Materials and Methods:**

Forty male soldiers (mean age 20.08 years) were recruited for the study. Prior to their first shooting practice, PTA and TEOAE were recorded. After 15 minutes and one week post- practice PTA and TEOAE were compared.

**Results:**

Immediately after shooting practice significant differences in PTA at 500, 1000, and 4000 Hz were observed for the right ear and no significant difference at any frequency for the left ear. There was a significant difference in the amplitude of TEOAE 15 minutes after shooting practice at 500, 1000, 2000, 3000, and 4000 Hz in the right ear, while for the left ear the difference was significant at 1000 and 2000 Hz. One week after exposure a significant difference at 500 and 4000 Hz was found only in the right ear and a significant difference in the amplitude of TEOAE was observed at 500, 1000, 2000, 3000, and 4000 Hz.

**Conclusions:**

Even exposure lower than permissible levels may lead to acoustic trauma. TEOAE is more sensitive than PTA in detecting early hearing loss after military shooting exercises. Hearing protection equipment and appropriate surveillance programs are recommended.

## 1. Background

Impulse noise (IN) is a transient noise which consists of one or more bursts of sound energy. Military personnel are usually exposed to high levels of IN and can suffer from a wide range of IN effects on hearing including acoustic trauma (1-5). It has been shown that auditory symptoms including hearing loss and tinnitus increase during military service (6). More than 28% of US and 20- 30% of army personnel suffer from hearing loss (7, 8). Acoustic trauma occurs as result of exposure to IN. This will cause sudden sensorineural hearing loss through mechanical damage of the structures of the middle or inner ear (mainly hair cell damage) (9). These pathologic changes may be transitory or permanent which may result in temporary or permanent threshold shift (TTS vs. PTS). TTS is a hearing damage which usually occurs after transient exposure to a very loud noise and is reversible within 24 hours after exposure. Continuous or severe exposure to loud noise can cause PTS which is an irreversible hearing loss in high frequencies (1-5).

Since military environments expose army personnel to very high levels of IN (10-13), it is of utmost importance to detect early temporary or permanent hearing damage in soldiers and to identify their susceptibility to noise exposure in order to provide better protection and prevention (10, 11, 13). Otoacoustic emission (OAE) provides the possibility of early diagnosis of noise induced hearing loss and is considered to be a very promising tool to detect mild hearing loss in exposed subjects (14-16) compared to pure tone audiometry (PTA) (17-21). Transiently evoked otoacoustic emission (TEOAE) is a sensitive, objective and frequency specific audiometric test for evaluating early hair cell damage (10, 11, 13-17).

## 2. Objectives

The aim of this study was to evaluate the effect of low level IN produced by gunfire in 40 military personnel after their first firing practice and to compare the sensitivity of TEOAE and pure tone audiometry in detection of TTS and PTS following firing practice.

## 3. Materials and Methods

Forty male soldiers (mean age 20.08 years) were recruited for this study to be evaluated following their first firing practice. Those with any current or past history of hearing problems, history of systemic diseases, history of using ototoxic drugs, previous excessive exposure to noise, ototoxic chemicals or abnormal hearing results (hearing threshold more than 20 dB) were not included in the study. The Institutional Review Board of Tehran University of Medical Sciences approved the protocol of the study. The training exercise consisted of one round of 10 single shots and one round of 10 continuous shots using a Kalashnikov rifle in recumbent position. The soldiers did not use hearing protectors during training. All were right-handed. The C-weighted peak sound pressure level (SPL) (LCs) and an equivalent continuous A-weighted SPL (LAs ) during shooting were determined using a real-time frequency analyzer (type 2131 B&K with expansion unit type 5765 B&K). Frequency analysis was also performed for 500, 1000, 2000, 4000, and 8000 Hz frequencies.

Before firing, 15 minutes post-firing, and one week after the practice all subjects completed a questionnaire and underwent otoscopic assessment and PTA as well as TEOAE were recorded. PTA was recorded in an acoustic cabin using a Danflex DA65 audiometer at the range of frequency from 0.5 to 8 kHz. Otoacoustic emissions were recorded using an ILO 292 Echoport version 5.0 (Otodynamics Ltd). TEOAE recordings were collected for every subject at stimuli levels of 80 ± 2 dB SPL using 80 ms duration clicks in a nonlinear pattern. An artifact rejection level of 4.6 mPa (47.3 dB SPL) was used throughout the recording session. Each response was windowed from 2.5 to 20 ms post-stimulus and band pass filtered from 0.5 to 6 kHz. Values of TEOAE and signal to noise ratio (SNR) were examined in 1 kHz bands from 1 to 5 kHz. Only emissions with an amplitude of at least two standard deviations above the noise were accented as a true OAE. A threshold shift which was detected 15 minutes after exposure and reversed after one week was considered as TTS; a threshold shift which did not recover at one-week postexposure measurements was considered PTS. The effect of exposure to the impulse noise was evaluated using t-Student test comparing pre-exposure measurements to 15-minute and one-week post-exposure measurements for PTA, TEOAE, and SNR.

## 4. Results

Forty male years recruits (80 ears) with a mean ± SD age of 20.08 ± 2.61 were studied. All 40 were right-handed and held the weapon with their dominant arm. The mean duration of exposure to gunshot noise was 190 seconds. The noise level for single-shot round for the impulse A-weighted sound level LA_Im_ dB SPL was between 105 and 113 (mean 108.3) for the right ear and between 100 and 110 (mean 105.7) for the left ear. The impulse C-weighted sound level LC_Im_ dB SPL was between 104 and 112 (mean 107.6) for the right ear and between 102 and 109 (mean 105.9) for the left one after the single-shot round. The broadband noise analysis for the single-shot round showed that the highest noise levels were found in the frequencies 2 KHz and 4 KHz near the left and the right ear (Table 1). For continuous-shot round the sound pressure for the impulse A-weighted sound level LAs, dB SPL was between 108 and 115 (mean 110.5) for the right ear and between 105 and 112 (mean 109.2) for the left ear. The impulse C-weighted sound level LCs, dB SPL was between 109 and 117 (mean 111.4) for the right ear and between 106 and 114 (mean 110.1) for the left ear after the continuous-shot round. The broadband noise analysis for the continuous-shot round showed that the highest noise levels were found in the frequencies 1 KHz and 2 KHz near the left and the right ear (Table 1).

**Table 1.** Mean Sound Pressure Level and the Broadband Noise Analysis after Shooting Practice.

	LAIm (dB SPL)	LCIm (dB SPL)	125 Hz	250 Hz	500 Hz	1 KHz	2KHz	4KHz	8KHz
Single-shot round for the right ear	108.3	107.6	73.6	86.2	92.8	94.9	106.1	101.8	97.8
Single-shot round for the left ear	105.7	105.9	74.5	89.1	91.7	96.8	103.5	98.1	91.3
Continuous-shot round for the right ear	110.5	111.4	79.9	92.4	102.2	104.9	105.9	102	97.4
Continuous-shot round for the left ear	109.2	110.1	79.6	91.6	100.1	102.5	105.3	101.4	97.1

Pre-exposure assessment showed that all participants had normal PTA as well as TEOAE. Fifteen minutes after exposure, 20 subjects (50%) had abnormal PTAs. Comparison between PTA results in different frequencies before and 15 minutes after exposure (Table 2) showed that there was a significant difference at 500 Hz (*P* < 0.001), 1000 Hz (*P* = 0.04), and 4000 Hz (*P* = 0.029)in the right ear. In the left ear, however, there was no significant difference at any frequency. Whole wave reproducibility (WWR) of the right ear and WWR of the left ear 15 minutes after exposure were significantly different from before exposure. There was a significant difference in the amplitude of TEOAE 15 minutes after shooting at 500, 1000, 2000, 3000, and 4000 Hz in the right ear with a mean change of 2.5 dB SPL at 500 Hz (*P* <0.02), 2.4 dB SPL at 1000 Hz (*P* <0.02), 2.6 dB SPL at 2000 Hz (*P* <0.03), 3.1 dB SPL at 3000 Hz (*P* <0.03) and 5.1 dB SPL at 4000 Hz (*P* <0.01). Decrease in TEOAE amplitude 15 minutes after exposure was significant at 1000 Hz (4.3 dB SPL, *P* = 0.03) and 2000 Hz (2.6 dB SPL, *P* = 0.04) for the left ear (Table 3).

**Table 2.** Comparison Between PTA Results in Different Frequencies Before and 15 Minutes After Exposure.

Tested Frequencies	Mean ± SD 15 min after exposure	Mean ± SD before exposure	Mean difference	*P* -Value
500 Hz (right ear)	2.5±4.9	0.2±4.3	2.3	< 0.001
1 KHZ(right ear)	-2.7±4.7	-3.6±4.1	0.96	0.040
2 KHz (right ear)	-0.8±4.1	-1±4.5	-0.19	0.574
4 KHz(right ear)	12.1±8.3	10.6±6.9	1.5	0.029
8 KHz(right ear)	11.9±7.5	1.8±7	1.15	0.313
500 Hz (left ear)	1.9±4.5	0.9±4.7	0.96	0.134
1 KHZ(left ear)	-3.8±5.4	-4.4±5.2	0.57	0.327
2 KHz (left ear)	0.4±5.5	0.4±5.8	0	0.999
4 KHz(left ear)	11.3±7.7	10.8±9.5	0.57	0.559
8 KHz(left ear)	11.9±9.5	10.2±12.4	1.7	0.185

**Table 3.** Comparison Between TEOAE Results in Different Frequencies Before and 15 Minutes After Exposure.

Tested Frequencies	Mean±SD before exposure	Mean±SD 15 min after exposure	Mean difference	*P* -Value
WWR(right ear)	75.3±21.4	66.4±26.2	8.91	0.019
SNR 0.5KHz(right ear)	11.5±7.5	9±7.4	2.5	0.019
SNR 1KHz(right ear)	11.5±7.5	9.1±7.4	2.4	0.019
SNR 2KHz (right ear)	12.3±5.6	9.7±5.9	2.6	0.029
SNR 3KHz(right ear)	8.2±5.4	5.1±5.8	3.1	0.029
SNR 4KHz(right ear)	7.4±5.1	2.3±4.8	5.1	0.008
WWR(left ear)	69.5±24.4	62.1±33.6	7.4	0.026
SNR 0.5KHz(left ear)	10.1±5.8	8.7±5.9	0.4	0.082
SNR 1KHz(left ear)	10.2±5.8	5.9±5.9	4.3	0.012
SNR 2KHz(left ear)	10.3±6.1	7.7±6.7	2.6	0.041
SNR 3KHz(left ear)	6.7±4.7	6.2±5.7	0.48	0.605
SNR 4KHz(left ear)	4.5±5.7	4.6±5.6	0.04	0.962

One week after exposure, 15 subjects (37.5%) had abnormal PTAs. Comparison between PTA results in different frequencies before and one week after exposure (Table 4) showed that there was a significant difference at 500 and 4000 Hz (*P* < 0.05) in the right ear, however significant difference at any frequency was not observed in the left ear. WWR of the right ear one week after exposure was significantly different from before exposure (*P* =0.023); however, the difference was not significant for the left ear. There was a significant difference in the amplitude of TEOAE one week after shooting at 500, 1000, 2000, 3000, and 4000 Hz in the right ear with a mean change of 2.1 dB SPL at 500 Hz (*P* < 0.03), 2.2 dB SPL at 1000 Hz (*P* < 0.02), 2.4 dB SPL at 2000 Hz (*P* < 0.03), 2.9 dB SPL at 3000 Hz (*P* < 0.03) and 4.9 dB SPL at 4000 Hz (*P* < 0.01) both for the whole response as well as for 1/2-octave band responses. Decrease in TEOAE amplitude for the left ear at one week was present at 1000 Hz (1.3 dB SPL) and 2000 Hz (0.4 dB SPL) but as mentioned above these differences were not statistically significant (*P* > 0.05) (Table 5).

**Table 4.** Comparison Between PTA Results in Different Frequencies Before and One Week After Exposure.

Tested Frequencies	Mean±SD 15 minutes after exposure	Mean±SD before exposure	Mean difference	*P* -Value
500 Hz (right ear)	2.1±4.6	0.2±4.3	9.1	0.011
1 KHZ(right ear)	-2.9±4.8	-3.6±4.3	0.7	0.145
2 KHz (right ear)	-1±4.5	-1±4.5	0	0.574
4 KHz(right ear)	11.9±8.9	10.6±6.9	1.3	0.031
8 KHz(right ear)	1.9±7.0	10.8±7	0.15	0.563
500 Hz (left ear)	1.4±4.6	0.9±4.7	056	0.237
1 KHZ(left ear)	-4.2±5	-4.4±5.2	0.14	0.444
2 KHz (left ear)	0.4±5.1	0.4±5.8	0	1.287
4 KHz(left ear)	11.1±7.4	10.8±9.5	0.34	0.559
8 KHz(left ear)	11.1±9.7	10.2±12.4	0.9	0.344

**Table 5.** Comparison Between TEOAE Results in Different Frequencies Before and One Week After Exposure.

Tested Frequencies	Mean±SD before exposure	Mean±SD one week after exposure	Mean difference	*P*- Value
WWR(right ear)	75.3±21.4	68.3±26.8	6.83	0.022
SNR 0.5KHz(right ear)	11.5±7.5	9.4±7.9	2.1	0.029
SNR 1KHz(right ear)	11.5±7.5	9.3±7	2.2	0.019
SNR 2KHz (right ear)	12.3±5.6	9.9±6.1	2.4	0.029
SNR 3KHz(right ear)	8.2±5.4	5.3±5.8	2.9	0.029
SNR 4KHz(right ear)	7.4±5.1	2.5±4.3	4.9	0.008
WWR(left ear)	69.5±24.4	64.1±33.9	5.4	0.054
SNR 0.5KHz(left ear)	10.1±5.8	8.9±5.9	0.2	0.198
SNR 1KHz(left ear)	10.2±5.8	8.9±4.8	1.3	0.052
SNR 2KHz(left ear)	10.3±6.1	9.9±6.7	0.4	0.570
SNR 3KHz(left ear)	6.7±4.7	6.4±5.7	0.22	0.975
SNR 4KHz(left ear)	4.5±5.7	4.4±6.6	0.01	0.992

Immediately after exposure, four acquired clinical deafness of the right ear and two acquired clinical deafness of both ears. After one week, 4 had clinical hearing loss of the right ear and 1 had clinical hearing loss of the both ears. After shooting, extra-auditory symptoms included tinnitus (25 subjects), dizziness (16 subjects), discomfort when exposed to loud sounds (29 subjects) and problems in speech discrimination (30 subjects). In one subject, rupture of the tympanic membrane was evident with a 30-dB decrease in all frequencies at audiometry. Three participants had earache, inflammation and limitation of tympanic membrane movement and were treated. However, examination of the pharynx and tonsils was normal. Of the aforementioned three subjects, one had clinical hearing loss. These problems completely resolved after one week.

## 5. Discussion

Our study noted that exposure to lower levels of IN can disturb cochlear function in which TEOAE is more sensitive than PTA. Also indices of TEOAE demonstrated other than 3000-6000 Hz, low frequency hearing spectrum (500-2000 Hz) may be involved. Peak level of impulse noise varies according to different standards. For example NATO has notified a value limit of 160 dB for military noises (22). The Occupational Safety and Health Administration (OSHA) criteria for unprotected occupational noise exposure considers the “peak” (not time-averaged) unweighted sound level of 140 dB, (C) limit to be more practical. Excessive noise exposure above the permissible limit requires noise exposure reduction (23). In gunfire practice , our study peak exposure level was lower than mentioned standards.

The small-caliber weapons have been considered to produce a peak level of 132-165 dB with a frequency spectrum between 150 to 2500 Hz (22-24). Even explosion noise of gunfire can reach up to 170 dB A (1, 5). In Nipapan *et al*. (25) study gunfire practice produced IN up to 127 dBA. After 3 days of exposure they found only one person with acoustic trauma. Also an exposure of 119- 127 dB SPL was seen in the results of Flamme *et al*.(26). They stated that small-caliber guns with long barrels have low auditory risks. Pawlaczyk *et al*.(27) noted that after 3-4 exposures to IN as high as 154 dB significant reduction of TEOAE levels were seen. Nonetheless we found that after exposure to 106 to 117 dB SPL permanent threshold shift occurred in 37.5% of participants. According to ISO 1999 the determination of threshold shift due to continuous or impulse noise is based on A-weighted equivalent continuous sound pressure level on equal energy basis (28). Nevertheless the most important parameter of impulse noise regard to hearing effects is C-weighted or unweighted peak sound pressure levels (29). In our study both C and A- weighted levels were measured, but analysis was conducted on the basis of C-weighted level.

Typically, in audiogram the first sign due to occupational noise exposure is change at 3,000, 4,000, or 6,000 Hz, with recovery at 8,000 Hz (30). In the case of IN higher prevalence of 3000 to 6000 Hz was stated by Mrena *et al*. (31), although in Norway the effect of IN on 4000 and 8000 Hz was the same (32). Involvement of low frequencies is less acceptable (33), but interestingly we saw early and late changes in 500, 1000 and 2000 Hz in TEOAE .This was similar to the results of Pawlaczyk *et al*. (27). Who stated that the greatest change was seen in amplitudes at 1500 Hz and 2000 Hz.

In this study, temporary threshold shift was evident among the subjects and permanent threshold shift was documented in the right ear. However, no significant difference existed in the number of subjects with clinical hearing loss before, immediately after and one week after practice which represents the necessity of occupational hearing surveillance by means of audiologic tests. Furthermore all of our subjects were right-handed and their right ears were positioned closer to the source of noise during shooting. This may explain the greater changes of hearing results in the right ears. In other studies involvement of right ear is more common^25^. The most common clinical finding after exposure to IN is tinnitus. Current consensus is that the functional integrity of outer hair cells and nerve fibers is disrupted (1, 9). However, the pathophysiology of subjective tinnitus is not clear. Damaged hair cells make repeated spontaneous emissions to the central auditory pathway. It is possible that misunderstanding of this signal as real sound causes the tinnitus (34). In our study more than 60% experienced post-exposure tinnitus. Jokitulppo (35) stated that exposure to IN has a correlation to tinnitus. The occurrence of noise related auditory changes depends on exposure type (e.g., sound level, duration, type of noise, and frequency), as well as personal factors (e.g., susceptibility to noise, age, smoking, prior history of hearing/ear damage) (36, 37).

According to our findings exposure to IN produced by small caliber types of guns is clinically important and personal protection and occupational health surveillance should be considered for all military personnel who fire guns Also assessment of hearing should be performed at the end of the training course for better evaluation of permanent changes. We also recommend that hearing assessment be performed for the frequencies that we did not assess in this study and particularly for higher frequencies, as reduced TEOAE levels in these frequencies may be the earliest sign of hearing loss (38, 39). These findings can guide us in developing better hearing protection aids.
